# Serum Cannabinoid 24 h and 1 Week Steady State Pharmacokinetic Assessment in Cats Using a CBD/CBDA Rich Hemp Paste

**DOI:** 10.3389/fvets.2022.895368

**Published:** 2022-07-22

**Authors:** Tongxin Wang, Alex Zakharov, Beatriz Gomez, Alex Lyubimov, Nathalie L. Trottier, Wayne S. Schwark, Joseph J. Wakshlag

**Affiliations:** ^1^Department of Animal Science, Cornell University, Ithaca, NY, United States; ^2^Department of Toxicology, University of Illinois at Chicago, Chicago, IL, United States; ^3^Department of Molecular Medicine, Cornell University, Ithaca, NY, United States; ^4^Department of Clinical Sciences, College of Veterinary Medicine, Cornell University, Ithaca, NY, United States

**Keywords:** cannabidiol, cannabidiolic acid, cannabinoid, feline, hemp (*Cannabis sativa* L.)

## Abstract

Hemp based cannabinoids have gained popularity in veterinary medicine due to the potential to treat pain, seizure disorders and dermatological maladies in dogs. Cat owners are also using hemp-based products for arthritis, anxiety and neoplastic disorders with no studies assessing hemp cannabinoids, namely cannabidiol efficacy, for such disorders. Initial twenty-four pharmacokinetic and chronic dosing serum concentration in cats are sparse. The aim of our study was to assess 8 cats physiological and 24 h and 1-week steady state pharmacokinetic response to a cannabidiol (CBD) and cannabidiolic acid (CBDA) rich hemp in a palatable oral paste. Using a standard dose of paste (6.4 mg/CBD + CBDA 5.3 mg/gram) across 8 cats weighing between 4.2 and 5.4 kg showed an average maximal concentration of CBD at 282.0 ± 149.4 ng/mL with a half-life of ~2.1 ± 1.1 h, and CBDA concentrations of 1,011.3 ± 495.4 ng/mL with a half-life of ~2.7 ± 1.4 h, showing superior absorption of CBDA. After twice daily dosing for 1 week the serum concentrations 6 h after a morning dosing showed that the acidic forms of the cannabinoids were approximately double the concentration of the non-acidic forms like CBD and Δ9- tetrahydrocannabinol (THC). The results of this study compared to two other recent studies suggest that the absorption in this specific paste product may be superior to oil bases used previously, and show that the acidic forms of cannabinoids appear to be absorbed better than the non-acidic forms. More importantly, physical and behavioral examinations every morning after dosing showed no adverse events related to neurological function or behavioral alterations. In addition, bloodwork after 1 week of treatment showed no clinically significant serum biochemical alterations as a reflection of hepatic and renal function all remaining within the reference ranges set by the diagnostic laboratory suggesting that short-term treatment was safe.

## Introduction

Cannabinoids have gained significant interest in the biomedical field in recent decades. The fast-growing hemp industry for textile manufacturing worldwide and the increasing legalization of marijuana, have contributed to an expansion in commercial availability of cannabinoid products (1). Both hemp and marijuana plants fall under the same genus *Cannabis*, but belongs to different cultivated varieties namely *Cannabis sativa* L. and *Cannabis indica* L., respectively (2, 3). Both varieties share a complex mixture of more than 120 cannabinoid compounds (4). Of these cannabinoids the cannabis plant produces predominantly cannabidiolic acid (CBDA) and tetrahydrocannabinolic acid (THCA), which during heat extraction becomes decarboxylated to cannabidiol (CBD) and Δ9-tetrahydrocannabinol (THC). Consequently, CBD and THC have received the most interest for their potential medicinal applications (5).

Both CBD and THC are bioactive molecules that act as agonists at receptors directly affecting the central nervous system in humans and animal models (6). Their potential use as nutraceuticals or even pharmaceutical products has been supported by several studies including CBD effectiveness for epilepsy treatment in young children (7), and protective effects on stress vulnerability and neurotoxicity for addiction disorders treatment (8). Recently, CBD and THC were shown to have anti-inflammatory, anti-emetic, anti-convulsant and anti-carcinogenic effects (9).

The use of cannabinoids in companion animal medicine is an emerging field of interest with limited studies available. In dogs with idiopathic epilepsy, CBD appears to alleviate seizure frequency (10). Brioschi et al. showed that CBD relieved osteoarthritic pain and improved life quality in dogs, allowing a reduction of other drugs' dosage and thus minimizing their potential side effects (11). Wakshlag et al. tested the absorption and retention of oral administration of CBD-rich hemp and demonstrated its safe use in dogs (12). Additionally, three other studies have shown beneficial effects of CBD-rich hemp nutraceuticals for treatment of canine osteoarthritis (11, 13, 14). Data on cannabinoids absorption and retention, and their clinical efficacy in cats is lacking. So far, only two studies have reported on the safety and tolerability of CBD and THC or CBD/CBDA rich hemp in healthy cats (15, 16). Pharmacokinetics of cannabinoids from CBD-rich hemp and their safety in the feline model are essential before providing any guidance on nutraceutical use of these products or other isolated cannabinoids for their potential use in feline medicine.

Prior pharmacokinetic data in dogs reveal that the native form of CBD and THC, which are CBDA and THCA, are absorbed and retained better than their decarboxylated forms (12, 17). Yet the medicinal value of these native cannabinoids from hemp plants are in their infancy in veterinary medicine. The native carboxylated molecules found in the plant are often seen in edible forms since heat extraction, vaping or smoking of *Cannabis* leads to decarboxylation of CBDA and THCA. Therefore, CBD and THC have been the primary cannabinoids of interest in human medicine research with few investigations into the absorption and retention of CBDA or THCA, both of which are non-psychotropic, unlike THC (9, 10).

Considering the absorption and retention kinetics of CBDA and THCA are unknown and may have potential value medicinally, the objective of this study was to perform 24-h pharmacokinetics of a CBD/CBDA-rich hemp extract containing minor cannabinoids including THC, THCA, cannabigerol (CBG) and cannabigerolic acid (CBGA) in cats. Additionally, cats were treated for a 1-week duration using a twice daily dosing of this CBD/CBDA-rich hemp extract to assess any adverse effects including behavioral and neurological as well as changes in complete blood counts and serum chemistry as measures of short-term treatment safety.

## Methods

### Animals and Animal Health

Eight cats from a contract research laboratory (Clinvet Global, Sayre, PA) were utilized for this experiment after Institutional Animal Care and Use Committee approval of the proposed protocol. Cats were from an equal mix of spayed/neutered male and female cats (4M, 4F). For the 24-h pharmacokinetic analysis each cat was provided a palatable paste formulation with ~6.4 mg/g CBD, 5.3 mg/g CBDA, 0.25 mg/g THC, 0.17 mg/g THCA, 0.13 mg/g CBG and 0.13 mg/g CBGA as measured by third party analysis prior to study initiation. The cat population ranged from 4.2 to 5.4 kg (mean weight 4.7 kg) and all cats were dosed based on practical clinical application which was 1 g of paste twice daily for 6 days after the initial 24-h pharmacokinetic assessment with treatment at 7 am and 4 pm each day. The cats were fasted prior to administration for nearly 15 h, and all cats were observed to accept and eat the paste without any negative behaviors and were fed their normal daily meal ~1 h after oral paste consumption. The base of the paste was a proprietary mix of soy oil, dextrose, dried chicken liver, silica, inulin, polysorbate 60, potassium sorbate, sorbic acid, saccharin, mixed tocopherols, methylparaben, and propylparaben in descending order of volume with 15% cannabinoid rich hemp oil emulsified with the product. Prior to enrollment in the study, all cats were deemed healthy and normal based on physical examination, complete blood count and serum chemistry assessment. During the 1-week trial, all cats were examined at the 1 and 4-h time point after the morning dosing for adverse events including diarrhea, vomiting, lethargy, somnolence, ataxia, or abnormal behavior. Blood was drawn (2 mL) via jugular venipuncture prior to the initial dose and then again at 0.5, 1, 2, 4, 8 and 24 h for a 7-point pharmacokinetic analysis. Complete blood counts, serum chemistry and serum cannabinoid concentrations were repeated at the end of day 7 treatment, 6 h after the morning treatment. Complete blood count data included white blood cells (WBC), hematocrit, hemoglobin, red blood cells (RBC), neutrophils, lymphocytes, platelets, monocytes, eosinophils, and basophils. Serum biochemistry analyses included sodium, potassium, chloride, magnesium, calcium, phosphorus, albumin, total protein, globulin, urea nitrogen, creatinine, alkaline phosphatase (ALP), alanine aminotransferase (ALT), aspartate amino transferase (AST), cholesterol, total bilirubin, glucose and gamma glutamyl transferase (GGT) with a focus of this report being renal and hepatic parameters. All blood parameters measured were performed at the Cornell University Diagnostic Laboratory.

### Serum Cannabinoids Analysis

Analysis was performed using an exploratory (fit-for-purpose) method for fast measurement of thirteen cannabinoids and their metabolites at the Toxicology Research Laboratory, University of Illinois at Chicago. The reference standards for CBD and CBDA were obtained from Restek Corporation (Bellefonte, PA) and all other reference and internal standards were obtained from Cerilliant Corporation (Round Rock, TX). Cannabinoids [CBD, CBDA, THC, THCA, cannabinol (CBN), cannabichromene (CBC), cannabigerol (CBG), and cannabigerolic acid (CBGA)] and their metabolites [11-hyrdoxytetrahydrocannabinol (11-OH-THC), 7-hyrdoxycannabidiol (7-OH-CBD), 7-nor-7-carboxycannabidiol (7-COOH-CBD), COOH-tetrahydrocannabinol (COOH-THC), and its glucuronide (COOH-THC-Glu)] concentration in cat serum was determined using high performance liquid chromatography with tandem mass spectrometry (LC-MS/MS) (Nexera X2 and LCMS 8050, Shimadzu Corp., Kyoto, Japan).

Cat serum (40 μL) was mixed with 20 μL of internal standards [100 ng/mL of CBD-d3, THC-d3, THCA-d3, 7-COOH-CBD-d3, 7-OH-CBD-d5, 11-OH-THC-d3, COOH-THC-d9, and COOH-THC-Glu-d3 in water:methanol (50:50)] in a 96-well plate. Proteins were precipitated and compounds were extracted by adding 100 μL of ice-cold acetonitrile to the samples, then vortexing for 1–2 min and centrifuging at 4,000 rpm for 10 min at 4°C. Supernatants (70 μL) were mixed with 70 μL of water in a different 96-well plate and centrifuged again. Processed samples (10 μL) were injected into Waters Atlantis T3 HPLC column (3 μm 2.1 × 50 mm) with a guard cartridge (Waters VanGuard Atlantis T3) coupled to LC-MS/MS. The column was equilibrated with mobile phase A (0.1% formic acid in water) and mobile phase B (acetonitrile) at 50% B. The compounds were eluted by a linear gradient from 50 to 95% B over 6 min, and then held at 95% B for 1 min. Subsequently, the column was re-equilibrated at initial composition for 1 min at a flow rate of 0.3 mL/min. The autosampler and column temperature were set a 4 and 30°C, respectively. The compounds were detected in electrospray ionization positive and/or negative mode as described in the Supplementary Table 1. Interface voltage and temperature were 4 kV and 300°C, respectively. Desolvation line and heat block temperatures were 250 and 400°C, respectively. Nebulizing, heating, and drying gas flow were 2.7, 5, and 5 L/min, respectively. For further information on the calibration curve range in cat serum, internal standards utilized and multiple reaction monitoring (MRM) transitions for each evaluation see Supplementary Table 1.

Concentrations of cannabinoids were calculated by LabSolutions software (Shimadzu Corp., Kyoto, Japan) using a quadratic calibration curve with 1/c^2^ weighing based on relative response (peak area of cannabinoids/peak area of internal standards).

### Pharmacokinetics and Statistical Analysis

The 24-h non-compartmental pharmacokinetic analysis for each hemp-derived cannabinoid (CBD, CBDA, CBG, CBGA, Δ9-THC, THCA) was performed utilizing a commercial software system (PK solutions 2.0, Summit PK, Montrose, CO). Semi-log plots were utilized to determine linearity of the elimination profiles. The results generated were time to maximal concentrations (Tmax), maximum serum concentration (Cmax), elimination half-life (T $\frac{1}{2}$), area under the curve to the last time-point (AUC_0−24_), and mean residence time (MRT). The program predicts steady state average serum concentrations (Css Ave) based on the assumption that steady state levels are achieved after 5 half-lives with selected frequencies of administration. For the following metabolites: 7 OH-CBD, 7-COOH CBD, COOH-THC, and COOH-THC-Glu, only the maximal observed concentrations of single dose initially and the 1 week steady state serum concentrations are reported. All of these cannabinoid and metabolite values reported are the mean and the standard deviation across time points assessed and for week 1 serum concentrations at the midpoint of dosing. Serum concentrations of the midpoint-dosing interval were compared to the Css Ave using a Student *T*-test with a significant difference set at a *p*-value of 0.05. Results of the complete blood counts and serum biochemistry parameters were assessed for normality using a Shapiro Wilk's test. Most data were normally distributed and a paired Student's Test was used to assess changes over the week of treatment with a *p*-value set at < 0.05.

All statistical testing was performed with GraphPad Prism 6.0 (GraphPad Software Inc., LaJolla, CA) and graphs were generated by R Foundation for Statistical Computing (Vienna, Austria). Any result that was below the quantifiable limit for the respective cannabinoid was considered 0 for all graphing and representation of data.

## Results

**Figure 1:**
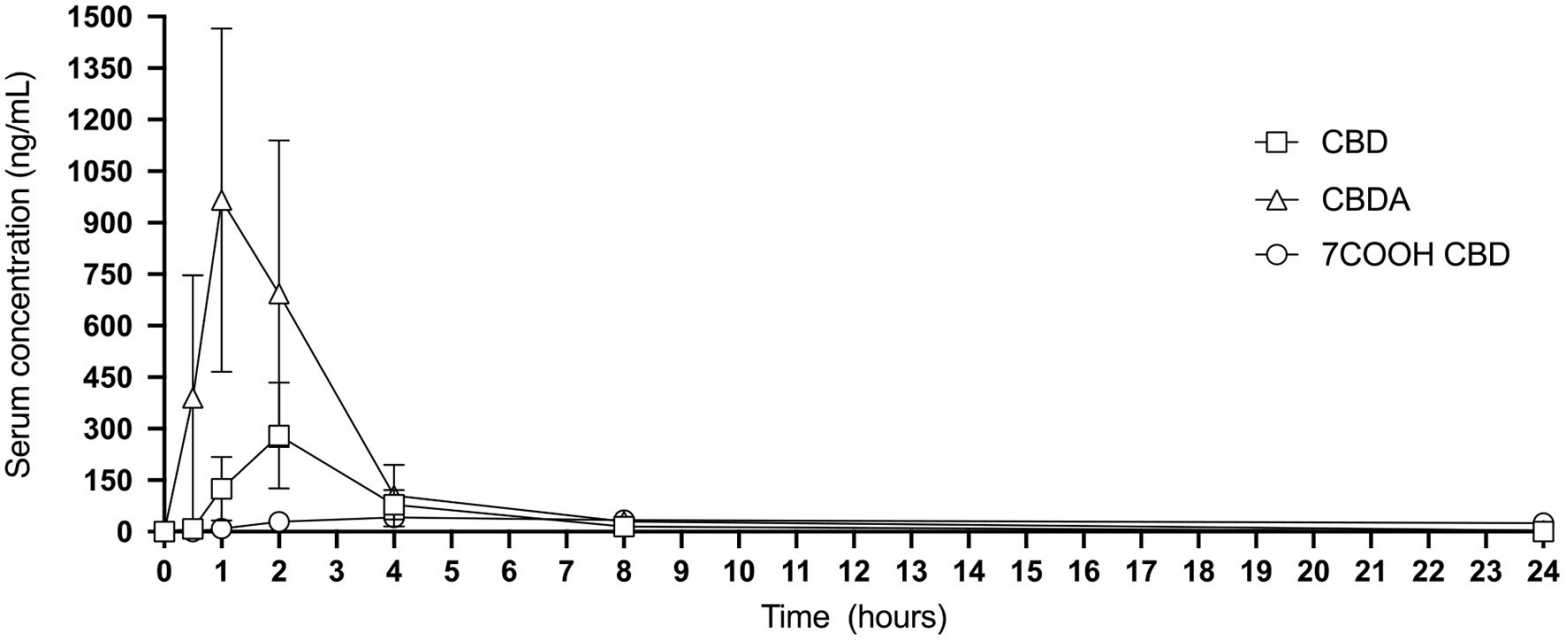
Twenty-four hour graphic representation of CBD, CBDA and metabolite 7-COOH-CBD serum concentrations (mean and standard deviation at each time point).

**Figure 2:**
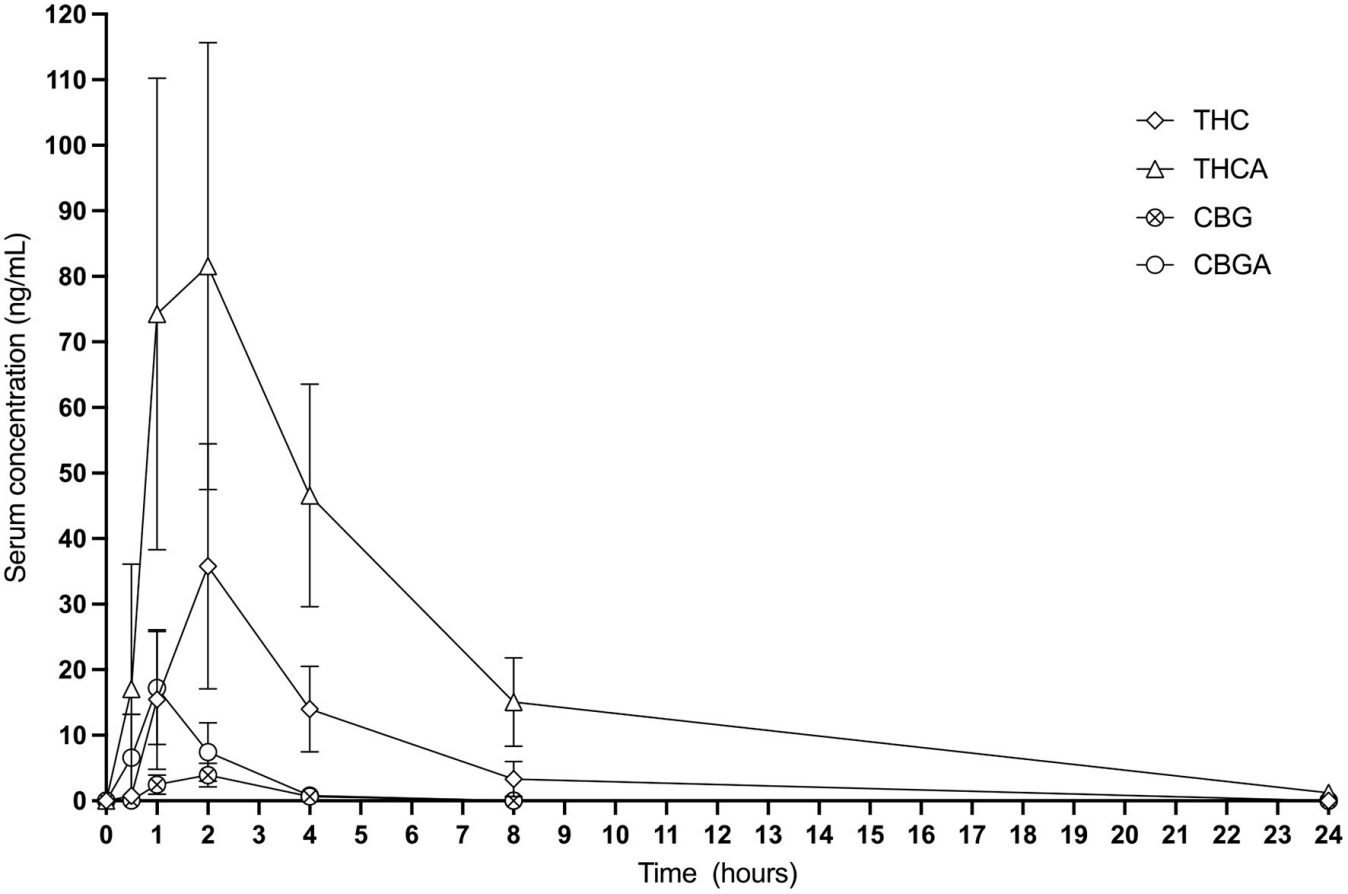
Twenty-four hour graphic representation of THC, THCA, CBG and CBGA serum concentrations (mean and standard deviation at each time point).

Calculable Cmax (ng/mL), Tmax (h), elimination T1/2 (h), AUC _0−24_ (ng-h/mL), MRT (h), and Css Ave means of CBD, CBDA, CBGA and THCA were obtained for all cats. Results for THC 24-h pharmacokinetics were only available for 7 of the 8 cats due to one cat only having three points above the lower limit of detection in the assay making generation of 24-h pharmacokinetics incalculable. Six of the 8 cats had concentrations above the lower limit for quantification of CBG for the 24-h pharmacokinetics. All other cannabinoids or metabolites (except for the metabolite 7-COOH-CBD) were either below the quantification limit or less than half of the population showed any appreciable concentration in the serum at any time point including 11-OH-THC, COOH-THC, COOH-THC-glu, 7-OH-CBD, CBN, and CBC. All mean and standard deviations across the population of cats for pharmacokinetics results are presented in Table 1. Figure 1 shows the 24-h serum concentrations curves of CBD, CBDA and 7-COOH-CBD and Figure 2 shows average serum concentrations of minor cannabinoids (THC, THCA, CBG, CBGA) across time points of the 24-h collection. The 7-COOH-CBD metabolite was found in all cats with a mean peak value of 41.4 ± 22.2 ng/mL at 4 h post dosing and the 1-week steady state concentration of 150.7 ± 50.1 ng/mL.

**Table 1:** Twenty-four hour pharmacokinetic analysis of hemp derived cannabinoids (CBD, CBDA, THC, THCA, CBG and CBGA).

	**Dose (mg/kg)**	**Cmax (ng/mL)**	**Tmax (h)**	**T1/2 el (h)**	**AUC 0—t (ng/mL)**	**MRT (h)**	**Css Ave (ng/mL)^**c**^**
CBD	1.37 ± 0.15	282.0 ± 149.4	2 ± 0	2.1 ± 1.1	908.5 ± 528.1	3.8 ± 1.0	102.1 ± 46.3
CBDA	1.13 ± 0.12	1,011.3 ± 495.4	1.6 ± 1.1	2.7 ± 1.4	2,638.7 ± 1,284.8	3.3 ±1.1	204.5 ± 87.8
THC^a^	0.05 ± 0.01	40.9 ± 12.9	2 ± 0	1.6 ± 0.4	156.5 ± 65.8	3.7 ± 0.4	11.9 ± 5.2
THCA	0.03 ± 0.003	87.9 ± 34.0	2 ± 0.9	3.8 ± 1.3	434.0 ± 229.1	5.5 + 2.1	43.3 ± 13.9
CBG^b^	0.03 ± 0.003	4.35 ± 1.0	2 ± 0	0.9 ± 0.1	11.4 ± 2.8	2.3 ± 0.1	2.3 ± 0.5
CBGA	0.03 ± 0.003	19.7 ± 5.5	1 ± 0.2	0.7 ± 0.1	34.1 ± 9.5	1.5 ± 0.2	4.8 ± 1.3

^a^*Represents 7 of 8 cats due to lack of time points having absorption above the lower level of detection to perform pharmacokinetic analysis in 1 cat. ^b^Represents 6 or 8 cats due to the lack of time points having adequate absorption above the lower level of detection to perform pharmacokinetic analysis in 2 cats. ^c^Css Ave = predicted steady state serum concentrations after 5 half-lives of twice daily dosing*.

**Figure 3:**
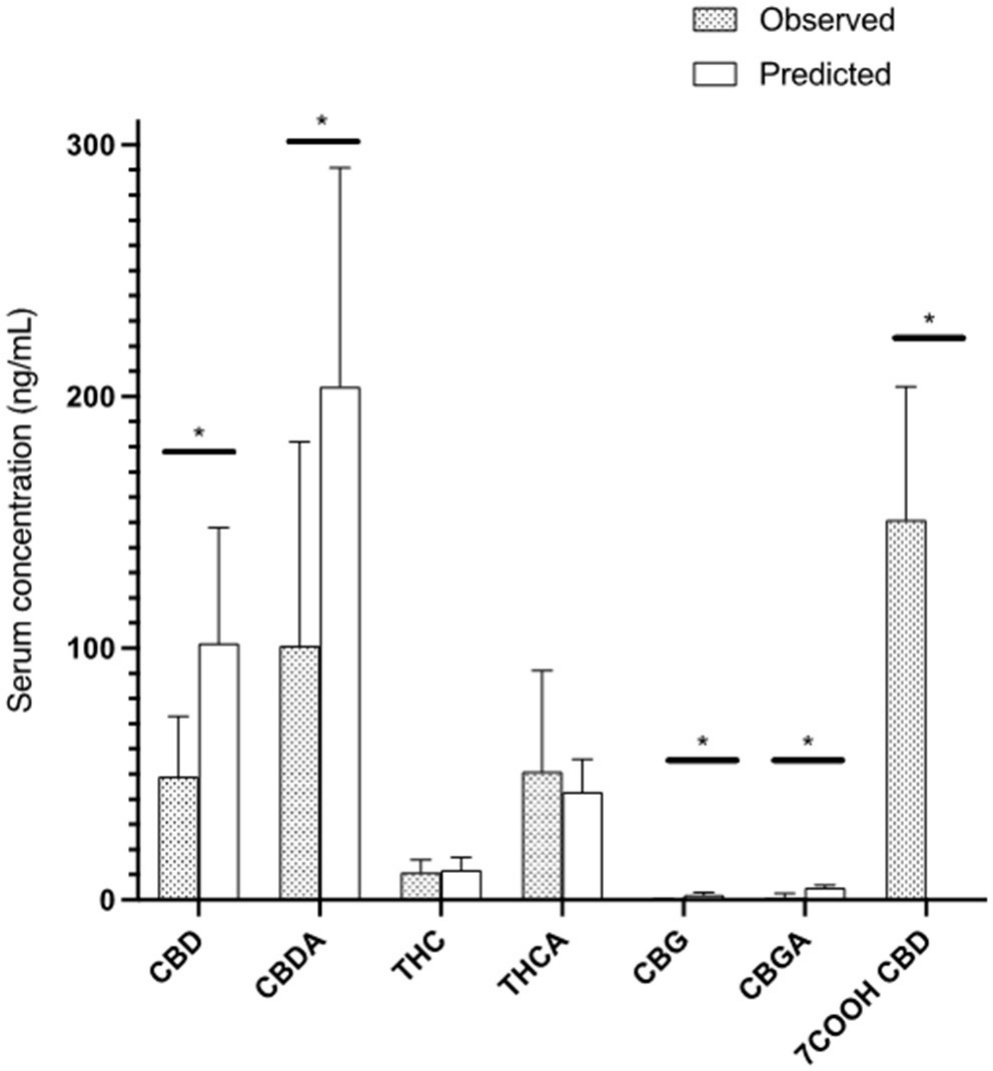
All cannabinoids at 6-h post 13th dose showing observed steady state concentrations in serum of cats compared to predicted steady state after 5 half-life of administration based on the 24-h pharmacokinetic analysis (Mean and standard deviation). * represents a statistically significant difference (*P* < 0.05).

One week 6 h post morning dosing concentrations were measured and compared to mean calculations. There was a significant difference (*p* < 0.05) between predicted Css Ave and actual serum concentrations for CBD (48.7 ± 24.0 ng/mL), CBDA (102.0 ± 81.2 ng/mL), CBG (0.3 ± 0.4 ng/mL) and CBGA (2.6 ± 2.8 ng/mL, while THC (11.4 ± 5.0 ng/mL; *p* = 0.76), THCA (50.6 ± 11.1 ng/mL) were similar to the predicted values in Table 1. For the CBD, CBDA, CBG and CBGA, the predicted concentration was higher than the actual concentration, while THC (*P* = 0.76) and THCA (*p* = 0.63) did not differ (Figure 3).

### Physical Examination Complete Blood Count and Selected Serum Chemistry Parameters

All cats were examined for adverse events after initial dosing and after morning dosing on each day of the trial. No adverse events regarding vomiting, diarrhea, hypersalivation or head shaking were noted at dosing, 1 h and 4 h after the morning dose over the week. Neurologically or behaviorally, no mydriasis, ataxia, somnolence, lethargy, or abnormal behavior were observed. Complete blood count assessments before and after treatment showed a mild decrease in white blood cell (*p* = 0.02), segmented neutrophil (*p* = 0.03), monocytes (*p* = 0.02), eosinophil (*p* = 0.04) and basophil concentrations (*p* = 0.03; Table 2) with no values at either the initiation of 1 week after treatment being outside of the normal reference range for any parameter.

**Table 2:** Complete blood counts and selected serum chemistry profile in cats before (pre) and 1 week (post) after oral administration of a CBD/CBDA rich hemp paste^a^.

**Item**	**Reference range**	**Pre**	**Post (1 week)**	***P*-value**
**Complete blood count**				
Hematocrit %	31–48	39.6 ± 3.1	37.4 ± 3.0	0.07
Hemoglobin (g/dL)	10.9–15.7	12.8 ± 0.9	11.7 ± 0.9	<0.01
RBC (mill/μL)^b^	6.9–10.1	9.4 ± 1.0	8.6 ± 0.9	<0.01
WBC (thous/μL)^c^	5.1–16.2	13.7 ± 2.7	11.4 ± 2.5	0.03
Seg. Neut. (thous/μL)	2.3–10.7	5.3 ± 1.7	5.1 ± 1.9	0.02
Lymphocytes (thous/μL)	1.2–6.8	5.1 ± 1.9	4.8 ± 1.4	0.62
Monocytes (thous/μL)	0.1–0.4	0.3 ± 0.1	0.2 ± 0.1	0.02
Eosinophils (thous/μL)	0.1–2.2	1.4 ± 0.6	1.1 ± 0.4	0.04
Basophils (thous/μL)	0.1–0.1	0.1 ± 0.1	0 ± 0	0.03
Platelet (thous/μL)	195–624	255 ± 52	324 ± 128	0.11
**Serum chemistry**				
Urea Nitrogen (mg/dL)	17–35	28 ± 3	27 ± 4	0.40
Creatinine (mg/dL)	0.8–2.1	1.5 ± 0.3	1.5 ± 0.3	0.14
Calcium (mg/dL)	9.0–11.3	10.4 ± 0.9	10.2 ± 1.0	0.18
Phosphorus (mg/dL)	2.6–5.5	4.9 ± 0.3	4.7 ± 0.2	0.05
Total Protein (g/dL)	6.6–8.4	7.3 ± 0.5	7.0 ± 0.5	<0.01
Albumin (g/dL)	3.2–4.3	3.8 ± 0.2	3.6 ± 0.2	<0.01
Globulin (g/dL)	2.9–4.7	3.5 ± 0.4	3.3 ± 0.3	0.02
Glucose (mg/dL)	71–182	75 ± 10	74 ± 7	0.84
ALT (U/L)^d^	26–109	66 ± 11	84 ± 18	<0.01
AST (U/L)^e^	17–48	26 ± 6	29 ± 5	0.18
ALP (U/L)^f^	11–49	35 ± 13	38 ± 16	0.44
Cholesterol (mg/dL)	101–223	157 ± 54	130 ± 43	<0.01

^a^*Values are mean ± standard deviation; ^b^Red blood cell; ^c^White blood cell; ^d^Alanine aminotransferase; ^e^Aspartate aminotransferase; ^f^Alkaline phosphatase*.

Serum renal and hepatic chemistry parameters showed a number of mild alterations. Serum albumin, globulin and total protein were decreased (*p* < 0.05) after 1 week of treatment (Table 2), and no parameters were outside the normal reference range at either time point. Serum creatinine, urea nitrogen and calcium did not differ between the time points, and phosphorus was lower (*p* < 0.01) after a week of treatment, but values did not fall outside of the reference range for any phosphorus values. There was an ALT elevation (*p* < 0.01) compared to baseline after 1 week of treatment, and no values were outside of the reference range prior to treatment or after 1 week of treatment. ALP and AST values did not differ, and two cats had elevations of ALP outside of the reference range prior to initiation of treatment and only 1 remained just above the reference range 1 week after treatment. Serum cholesterol was lower (*p* < 0.01) after the 1 week of treatment, and none of the values before or after treatment were outside of the reference range. GGT and total bilirubin are not represented on the tables as these values were 0 for all cats pre and post treatment.

## Discussion

Cannabinoids, notably THC and CBD have been the focus of studies in the pharmaceutical industry (15). Additional cannabinoids have received less attention regarding their safety and pharmacokinetics, yet are becoming a new focus for the *Cannabis* industry (18). Cannabinoids are synthesized in the *Cannabis* plant from the parent molecule CBGA (19), which is converted through synthase activities to both CBDA and THCA (20). Therefore, in the *Cannabis* plant, THC and CBD are primarily present in their carboxylic acid forms THCA and CBDA, respectively. These acidic cannabinoids, including CBGA (the parent molecule) are thermally unstable and are decarboxylated when exposed to UV light or heat (21). Supercritical carbon dioxide processing enables low heat extraction of the native molecules CBDA and THCA (16). This low heat extraction technology has allowed higher availability of the acidic forms in products for their potential use as therapeutics in this growing market. Sales of *Cannabis* related products including cannabis-derived herbal extracts for pets has increased by 1,000% between September 2016 and 2017 (19). The interest in cannabinoids for companion animals has expanded to include consideration of their potential benefits including modifying behavioral problems (15), alleviating seizure frequency for idiopathic epilepsy (10), and relieving osteoarthritic pain thus improving life quality for dogs (11).

As the demand for cannabinoid containing products increases, a better understanding of their safety, dosage, and pharmacokinetic properties is needed. There is a dearth of studies regarding dosage for efficacy and safety in pets, particularly cats. Deabold et al. (16) evaluated the pharmacokinetic and safety of CBD/CBDA-rich hemp on eight healthy cats and dogs over a 12-week period, with a twice daily fixed dosage of 2 mg/kg provided either in a fish oil base or soft chew, respectively. Throughout the course of the study, all cats had normal blood count and blood chemistry profiles, except for one cat who showed a persistently elevated serum ALT concentration. The main adverse effects were excessive licking and head shaking, with occurrence rates of 35.4 and 25.2%, respectively.

In a more recent study, Kulpa et al. assessed the safety and tolerability of cannabinoids in cats with 11 escalating dosages each separated with a minimum of a 3-day period (15). Both CBD and THC were tested in 2 treatment groups with dosage escalating from 2.8 to 30.5 mg/kg and 3.8 to 41.5 mg/kg, respectively. In addition, a mixture of CBD and THC was tested in one treatment group at escalating dosages of 1.2–13 mg/kg and 0.8–8.4 mg/kg, respectively. Adverse effects were noted within 24 h following the first dosage of 2.8 mg/kg CBD and 3.8 mg/kg THC, and for each dosage thereafter. Ataxia, membrana nictitans prolapse, and blepharospasm were associated with THC alone, while emesis, hypersalivation, and lethargy were associated with CBD alone. It is noteworthy to mention however, that statistical inference in that study was carried out only following the 11th dosage of CBD. In our study, CBD and THC were tested in combination with CBDA, THCA and CBGA, with a CBD and CBDA mean dosage of ~1.37 and 1.13 mg/kg, respectively; and 25-fold less amounts of CBG, CBGA, THC and THCA. All cats had normal blood counts and serum chemistry profiles within the reference range, with no observed physical or behavioral adverse effects, despite some decrease in the blood counts over the week. This may be related to the timing of blood drawing. Blood samples were taken at 7 am in the pre study phase, and ~1 pm in the afternoon for the 1 week follow up phase. Additionally, the serum chemistry alterations observed for blood proteins and cholesterol may have also been related to timing of blood draw, which is a limitation of this study as a placebo group was not included. Interestingly, serum ALT activity did increase in the population by a mean of ~18 U/L, similarly to the observations of Deabold et al. (16), hence unlike dogs where rises in ALP are observed, cats appear to have slight rises in the ALT hepatic enzyme. Markers of kidney compromise were unchanged, yet the more sensitive renal marker s-dimethylarginine was not examined in this acute dosing study. Of note in our population was a statistically significant decrease in RBC (and hemoglobin), neutrophil, eosinophil, and monocyte counts which were still within normal ranges for all cats. It is difficult to determine whether this was due to cannabinoids or the base paste ingredients; however, a prior study of long term dosing of a fish oil based CBD/CBDA-rich hemp extract similar in nature showed no differences in these parameters, suggesting a possible paste effect (16). A recent publication in guinea pigs undergoing lipopolysaccharide stimulation showed that using 50 mg/kg body weight orally resulted in a mild suppression of neutrophil counts in brochoalveolar lavage samples in a pulmonary model (22). Therefore, based on physical examination data, our dosage of 1.37 mg/kg of CBD and 1.13 mg/kg of CBDA compared to that of Deabold et al. (1 mg/kg of CBD and 1 mg/kg of CBDA) and Kulpa et al. (2.8 mg/kg of CBD) appears to be a range of safe tolerable dosage for cats with superior absorption (15, 16). Despite the presence of adverse behavioral effects in Deabold et al. which may potentially be related to the oil base, and in Kulpa et al. using CBD isolate alone in a medium chain triglyceride (MCT) base at ~12 mg/kg, this product shows promise regarding short term dosing safety (15, 16).

Our CBD dosage was slightly higher than that of Deabold et al., and the Cmax and AUC were five times higher (282.0 ± 149.4 vs. 43 ± 9 ng/mL, and 908.5 ± 528.1 vs. 164 ± 29, respectively) (Table 1), indicating greater CBD absorption in our study (16). In contrast, the other parameters including Tmax, t $\frac{1}{2}$, MRT were similar to those of Deabold et al. (16) (2 ± 0 vs. 2 ± 0.6 h, 2.1 ± 1.1 vs. 1.5 ± 0.1 h and 3.8 ± 1.0 vs. 3.5 ± 1.4 h, respectively). However, Kulpa et al. (15) reported 24-h pharmacokinetic data only following the ninth dose consisting of 25 mg/kg CBD or 34 mg/kg THC. Of these data, only values for Tmax were reported, ranging from 2 to 4 h with an average of 3.3 h. This high Tmax value is not surprising given the high CBD concentration tested in that study.

The higher apparent CBD absorption in our study may be due to either the matrix used to dose the cannabinoids or the presence of other cannabinoids in addition to CBD. Deabold et al. suggested that CBD absorption was higher in dogs compare to cats, in part due to the different matrix used (16). In their study, the 2 mg/kg dose of an equal mix of CBD and CBDA in fish oil resulted in a 24-h Cmax of ~43 ng/mL with a 2-h half-life for cats. Providing the same hemp extraction incorporated into a soft chew resulted in a Cmax over 250 ng/mL in dogs with a similar half-life, suggesting that the matrix can have a profound effect on absorption. The much higher CBD absorption in our study may be related to the matrix used which consisted of a food-based paste, composed in part of soy oil, dextrose, and dried chicken liver. The serum CBD concentration at the 2-h time in our study was similar to that of Kulpa et al. (280 vs. 256 ng/mL, respectively), despite that CBD dosage in Kulpa et al. was 18 times greater than our study (15). A pure medium chain triglyceride or fish oil-based matrix appeared to have caused inferior absorption in both studies (see Table 3 for comparative purposes). In most studies, animals are fasted before administration of CBD (Table 4), and thus the potential effects of feeding status prior to cannabinoid administration should not be ignored. In a recent meta-analysis, a greater absorption of CBD was reported for fed compared to fasted human individuals (24) (Table 4).

**Table 3:** Current studies of cannabinoids in cats.

	**Deabold et al**.	**Kulpa et al**.	**Wang et al**.
Study model	Dogs & cats	Cats	Cats
Cannabinoids	CBD, CBDA	CBD, THC, CBD & THC mixture	CBD, CBDA THC, THCA CBG, CBGA
Dosage (mg/kg)	2	Escalating: CBD: 2.8−30.5 THC: 3.8−41.5 Mix: 1.2 (CBD)/0.8 (THC)−13 (CBD)/8.4 (THC)	Fixed^a, b^ 1.37 mg/kg CBD 1.13 mg/kg CBDA 0.05 mg/g THC 0.03 mg/g THCA 0.03 mg/g CBG 0.03 mg/g CBGA
Pharmacokinetics	Yes	No	Yes
Serum chemistry liver markers	Yes	Yes	Yes

^a^*Each cat was given 1 gram of the mixture; ^b^The values were calculated based on per kg body weight, using average bodyweight of 4.7 kg*.

**Table 4:** Comparison of Serum pharmacokinetic of single oral dosing CBD oil in previous studies.

**References**	**Dosage of CBD (mg/kg)**	**Carrier of CBD**	**Fasted or not**	**Cmax (ng/mL)**	**Tmax (h)**	**T $\frac{1}{2}$ (h)**	**AUC (ng-h/mL)**	**MRT (h)**
**Cats**								
Deabold et al.^a^	1	Fish oil	Fasted	43 ± 9	2.0 ± 0.6	1.5 ± 0.1	164 ± 29	3.5 ± 1.4
**Dogs**								
Bartner et al. (30)^d^	5.77^j^	CBD- infused oil	Fasted	625.3 ± 164.3	NA^h^	199.7 ± 55.9	135.6 ± 46.3	217 ± 46
		Micro- encapsulated		346.3 ± 158.7		95.4 ± 29.2	98.0 ± 43.3	353 ± 48
		CBD-infused transdermal cream		74.3 ± 127.2		ND^i^	11.7 ± 18.9	490 ± 74
	11.54^j^	CBD-infused oil	Fasted	845.5 ± 262.2	NA^h^	127.5 ± 32.2	297.6 ± 112.8	298 ± 43
		Micro- encapsulated		578.1 ± 287.1		115.9 ± 88.6	162.8 ± 61.2	332 ± 73
		CBD-infused transdermal cream		277.6 ± 476		ND^i^	29.7 ± 29.6	464 ± 123
Gamble et al.^b^	1	Fish oil	Fasted	102 (61–132)	1.5 (1.0–2.0)	4.2 (3.8–6.8)	367 (183–437)	5.6 (4.2–9.1)
	4	Fish oil	Fasted	591 (389–905)	2.0 (1.0–2.0)	4.2 (3.8–4.8)	2,658 (1,753–3,048)	5.6 (5.1–7.0)
Deabold et al.^a^	1	Soft chews	Fasted	301 ± 63	1.4 ± 0.2	1.0 ± 0.2	1,297 ± 210	1.4 ± 0.3
Chicoine et al.^d^	2	CHE^c^	Fasted	213 (49)	2.1 (1.0)	2.5 (0.5)^e^	759 (335)^f^	346 (146)
	5	CHE^c^	Fasted	838 (304)	1.9 (0.6)	2.6 (0.4)^e^	2,935 (1,244)^f^	487 (182)
	10	CHE^c^	Fasted	1,868 (698)	2.3 (0.5)	2.3 (0.2)^e^	7,239 (2,393)^f^	588 (218)
Wakshlag et al.	1	MCT & sesame oil	Fed	145 ± 69	1.5 ± 0.5	4.1 ± 0.7	635 ± 399	5.2 ± 1.4
	1	Sunflower lecithin & sesame oil		124 ± 62	2.0 ± 1.1	4.4 ± 1.4	683 ± 146	6.5 ± 2.1
	1	Soft chews		226 ± 89	2.5 ± 1.2	3.8 ± 0.3	826 ± 74	5.3 ± 1.4
**Human**								
Silmore et al. (23)^g^	2.5	Sesame oil	Fasted	58.55 (60.52)	4.27 (23.25)	NA^h^	236.21 (46.05)	NA^h^
			Fed	218.51 (36.37)	3.28 (34.64)		1,015.45 (63.54)	

Cmax, maximum concentration; Tmax, time of maximum concentration; T1/2, half-life of elimination; AUC 0-t, area under the curve (time 0–24 h); MRT, median residence time. ^a^Mean ± standard error (SE) in parameters; ^b^Median and range in parameters; ^c^Cannabis-derived herbal extracts; ^d^Mean ± Standard deviation (SD) in parameters; ^e^T 1/2 phase, from 4 to 12 h post-dose; ^f^Dose-Adjusted AUC from 0 to 12 h (ng ^*^h/mL per mg/kg); ^g^The mean Cmax, Tmax, AUC 0-∞, and all corresponding standard deviations and coefficient of variation (CV%); ^h^Not available; ^i^Not determined; ^j^Calculated with average BW.

Hemp cannabinoid extract absorption kinetics may be dependent on the cannabinoid mixture being presented to the subject. Greater plasma cannabinoid and metabolite levels after administration of the CBD/THC mixture were found compare to CBD, or THC alone, suggesting a potential pharmacokinetic interaction between CBD and THC known as the “entourage effect” (15). In human seizure patients, it is becoming evident that when using whole plant hemp extract doses can be significantly reduced when compared to CBD isolate alone, further supporting this concept that minor cannabinoids or terpenes from the product may be inducing a synergistic effect with CBD (25). In our study, CBD was administered along with CBDA and in the presence of small concentrations of THC, THCA and CBGA as discussed earlier, and CBD absorption was far greater than that reported by Kulpa et al. (15). In addition, Pellesi et al. found greater absorption of THCA and CBDA compared to THC and CBD in humans (24). The higher absorption of those acidic forms illustrates their promising medicinal uses, as the unheated acidic forms will eliminate the psychoactive effects of THC (26).

So far, no study has been reported on the safety and tolerability for the acidic forms of cannabinoids in cats. Herein, CBDA, along with THCA and CBGA were administrated with dosage of 1.13, 0.05, 0.03 mg/kg, respectively. The addition of small quantity of minor cannabinoids did not lead to a higher Cmax for CBD (282 ng/mL), but may have resulted in a much greater CBDA mean concentration of 1,011 ng/mL. In fact, Anderson et al. suggested that cannabis extracts mixture can provide a natural vehicle to enhance plasma CBDA concentrations due to cannabinoid-cannabinoid interactions, thus potentially improving its absorption (27). The acidic forms of cannabinoids may become an important focus in the field and deserves further consideration to fully understand their functions and effectiveness, as tissue levels may not correspond to short lived serum levels.

Although there is safety data for chronic dosing extensive cannabinoid examination during twice daily dosing was not performed (16). Pharmacokinetic software allows for a serum steady state concentration to be predicted based on the assumption that steady state is achieved after 5 half-lives of administration. This should be similar to the serum concentrations that were measured 6 h after the 13th dose of administration on a twice daily regimen. We found that the serum concentrations were ~50% of what would be predicted to be the steady state for all cannabinoids except for THC and THCA. This suggests differential hepatic cannabinoid metabolism which is in its infancy regarding companion animals. Though speculative, it appears that there may be some induction of the cytochrome p450 system leading to higher than expected biliary or urinary excretion after a week of twice daily dosing. This is further supported with the 24-h pharmacokinetics results showing a maximal serum concentration of 7-COOH-CBD at ~40 ng/mL which increased to over 150 ng/mL by 1 week, suggesting an upregulation of hepatic metabolism. More importantly, these levels even after 1 week of dosing appear to be far less than what is observed as the major metabolite in rodents and humans (28, 29). These data and one other study on hepatic microsomes from a cat liver suggest alternative metabolism via the cytochrome p450 enzymatic pathways which has been proposed to be 4–6 hydroxylation or carboxylation (17). The physiological effects of these metabolites are currently unknown and there are no commercially available standards to evaluate metabolic disposal through the cytochrome p450 system to standardize evaluation of these metabolic byproducts.

In conclusion, this is the first comprehensive evaluation of hemp-derived cannabinoids from a specific product that is rich in both CBD and CBDA. Like observed in other species, there appears to be superior absorption of the acidic forms of cannabinoids when compared to their decarboxylated counterparts. Our observation of superior absorption of the acids is also evident for CBD in this specific formulation when compared to the two other studies utilizing CBD in either fish oil or MCT base (15, 16). More importantly with this superior absorption, we did not recognize any adverse events associated with a 1-week administration of the product. Nonetheless, findings from one CBD-rich hemp product cannot be extrapolated to other products due to the strain differences in cannabinoid production. Although we observed no clinically relevant alterations in complete blood count and serum chemistry parameters, it is still prudent to evaluate hepatic enzymes when deciding to treat due to a very mild rise in serum ALT.

## Data Availability Statement

The raw data supporting the conclusions of this article will be made available by the authors, without undue reservation.

## Ethics Statement

The animal study was reviewed and approved by Clinvet Global Institutional Animal Care and Use Committee.

## Author Contributions

JW was involved in the research design and implementation of the research plan. All authors were involved in the data analysis and preparation of this manuscript.

## Funding

The authors declare that this study received funding from Ellevet Sciences. The funder was not involved in the study design, collection, analysis, interpretation of data, the writing of this article or the decision to submit it for publication. JW and WS are paid consultants for Ellevet Sciences.

## Conflict of Interest

JW and WS are paid consultants for Ellevet Sciences. The remaining authors declare that the research was conducted in the absence of any commercial or financial relationships that could be construed as a potential conflict of interest.

## Publisher's Note

All claims expressed in this article are solely those of the authors and do not necessarily represent those of their affiliated organizations, or those of the publisher, the editors and the reviewers. Any product that may be evaluated in this article, or claim that may be made by its manufacturer, is not guaranteed or endorsed by the publisher.
