# Supplementary material for: Serum Cannabinoid 24 h and 1 Week Steady State Pharmacokinetic Assessment in Cats Using a CBD/CBDA Rich Hemp Paste
Source: Front Vet Sci. 2022 Jul 22;9:895368. doi: 10.3389/fvets.2022.895368 (PMC9355628; doi:10.3389/fvets.2022.895368)
Supplement: Supplementary file 1 [file Table_1.DOCX]

Supplemental Table 1. LC-MS evaluation parameters of detection, internal standards and calibration curve range (upper and lower limits of quantification).

| Reference Standard | | | Internal Standard | | Calibration Curve Range (ng/mL) |
| --- | --- | --- | --- | --- | --- |
| Name | Retention Time (min) | MRM (Polarity) | Name | MRM (Polarity) |  |
| CBD | 4.7 | 315>193 (+) | CBD-d3 | 318>196 (+) | 2.5 - 2,500 |
| CBDA | 4.4 | 359>219 (+) | CBD-d3 | 318>196 (+) | 1 – 2,500 |
| THC | 5.8 | 315>193 (+) | THC-d3 | 318>196 (+) | 1 - 1,000 |
| THCA | 6.3 | 357>313 (-) | THCA-d3 | 360>314 (-) | 0.25 - 1,000 |
| CBG | 4.6 | 317>193 (+) | CBD-d3 | 318>196 (+) | 0.5 - 1,000 |
| CBGA | 4.55 | 361>219 (+) | CBD-d3 | 318>196 (+) | 1 – 1,000 |
| CBC | 6.15 | 315>193 (+) | THC-d3 | 318>196 (+) | 2.5 - 1,000 |
| CBN | 5.4 | 311>223 (+) | CBD-d3 | 318>196 (+) | 0.5 - 1,000 |
| 7-COOH-CBD | 2.4 | 345>299 (+) | 7-COOH-CBD-d3 | 348>302 (+) | 1 - 1,000 |
| 7-OH-CBD | 2.5 | 331>201 (+) | 7-OH-CBD-d5 | 336>201 (+) | 10 - 1,000 |
| COOH-THC | 3.7 | 345>299 (+) | COOH-THC-d9 | 354>308 (+) | 0.5 - 500 |
| COOH-THC-Glu | 2.2 | 521>299 (+) | COOH-THC-Glu-d3 | 524>302 (+) | 0.5 - 500 |
| 11-OH-THC | 3.6 | 331>201 (+) | 11-OH-THC-d3 | 334>201 (+) | 5 - 1000 |
